# Associations Between Daily Symptoms and Pain Flares in Rheumatoid Arthritis: Case-Crossover mHealth Study

**DOI:** 10.2196/64889

**Published:** 2025-07-21

**Authors:** Ting-Chen Chloe Hsu, Belay B Yimer, Pauline Whelan, Christopher J Armitage, Katie Druce, John McBeth

**Affiliations:** 1 Centre for Musculoskeletal Research, The University of Manchester, Oxford Road, Manchester, M13 9PL, United Kingdom, 44 161-306-6000; 2 Centre for Health Informatics, Division of Informatics, Imaging & Data Sciences, The University of Manchester, Manchester, United Kingdom; 3 Manchester Centre for Health Psychology, The University of Manchester, Manchester, United Kingdom; 4 NIHR Greater Manchester Patient Safety Research Collaboration, The University of Manchester, Manchester, United Kingdom; 5 NIHR Manchester Musculoskeletal Biomedical Research Unit, Central Manchester University Hospitals NHS Foundation Trust, Manchester, United Kingdom; 6 School of Primary Care, Population Sciences and Medical Education, University of Southampton, Aldermoor Surgery, Aldermoor Close, Southampton, SO16 5ST, United Kingdom, +44 (0)23 8059 1759

**Keywords:** pain flares, mHealth, patient-generated data, rheumatoid arthritis, musculoskeletal, quality of life, smartphone, mobile health, wearable, sleep, physical activity

## Abstract

**Background:**

Mobile health (mHealth) technologies, such as smartphones and wearables, enable continuous assessments of individual health information. In chronic musculoskeletal conditions, pain flares, defined as periods of increased pain severity, often coincide with worsening disease activity and cause significant impacts on physical and emotional well-being. Using mHealth technologies can provide insights into individual pain patterns and associated factors.

**Objective:**

This study aims to characterize pain flares and identify associated factors in rheumatoid arthritis (RA) by (1) describing the frequency and duration of pain flares using progressively stringent definitions based on pain severity, and (2) exploring associations between pain flares and temporal changes in symptoms across emotional, cognitive, and behavioral domains.

**Methods:**

Our 30-day mHealth study collected daily pain severity and related symptoms (scores 1-5, higher are worse) via a smartphone app and passively recorded sleep and physical activity via a wrist-worn accelerometer. Pain flares were defined using a 5-point scale: (1) above average (AA): pain severity > personal median, (2) above threshold (AT): pain severity > 3, and (3) move above threshold (MAT): pain severity moves from 1, 2, 3 to 4 or 5. A case-crossover analysis compared within-person variations of daily symptoms across hazard (3 days before a pain flare) and control (3 days not preceding a pain flare) periods using mean and intraindividual standard deviation. Conditional logistic regression estimated the odds ratio (OR) for pain flare occurrence.

**Results:**

A total of 195 participants (160/195, 82.1% females; mean age 57.2 years; average years with RA: 11.3) contributed 5290 days of data. Of these, 88.7% (173/195) experienced at least 1 AA flare (median monthly rate 4, IQR 2.1-5). Nearly half experienced at least 1 AT or MAT flare (median monthly rate 2, IQR 1-4). These pain flares lasted 2 days (IQR 2-3) on average across definitions, with some extending up to 12 days. Worsening mood over 3 days was associated with a 2-fold increase in the likelihood of AT flares the following day (OR 2.04, IQR 1.06-3.94; *P*<.05). Greater variability in anxiety over 3 days increased the likelihood of both AT (OR 1.67, IQR 1.01-2.78; *P*<.05) and MAT flares (OR 1.82, IQR 1.08-3.07; *P*<.05). Similarly, greater variability in sleepiness (OR 1.89, IQR 1.03-3.47; *P*<.05) also increased the likelihood of AT flares. Sedentary time (%) consistently showed almost no influence across all definitions. Similarly, the simplest definition of AA demonstrated no significant associations across all symptoms.

**Conclusions:**

Pain flares were commonly observed in RA. Changes in sleep patterns and emotional distress were associated with pain flare occurrences. This analysis demonstrates the potential of daily mHealth data to identify pain flares, opening opportunities for timely monitoring and personalized management.

## Introduction

Digital technology plays a crucial role in modern medicine. It not only improves the essential routines and workflows of health care but also helps minimize accessibility barriers and time and location constraints, as well as streamlining health data management. As digital technology advances and becomes widely adopted, it is transforming our approach to addressing individual health needs by moving beyond traditional self-report methods and enabling more dynamic and continuous assessments. We can now collect patient information from multiple sources with greater time-varying details over extended periods. Mobile health (mHealth) devices, such as smartphones and wearables, allow patients to log symptoms actively through numerical scales or visual tools [1-5]. They can also passively capture high-resolution streams of health-related and contextual information, such as sleep, physical activity, or weather with minimal burden [3,5]. This enhanced capability of characterizing temporal changes in symptom patterns can deepen our understanding of individual health trajectories at a more granular level and open opportunities to improve disease management through more personalized strategies.

Individuals with chronic musculoskeletal conditions, such as rheumatoid arthritis (RA), typically experience persistent pain that requires continuous treatment and long-term management. Daily fluctuations in pain are common [6,7]. While not all fluctuations require clinical attention, periods of significantly increased pain severity, known as pain flares, have a greater impact and present clinical challenges due to their variability and unpredictability [8-10]. Pain flares often occur concurrently with escalated disease activity [11-13], but not every occurrence involves inflamed disease activity, nor does inflamed disease activity always result in escalated pain [11]. Additionally, pain flares exhibit considerable heterogeneity both within and across individuals and are influenced by a complex interplay of biopsychosocial, behavioral, and environmental factors [3,14-17]. For example, depression was identified as a significant contributing factor for more severe pain and greater pain fluctuations in rheumatic diseases [16]. Longer time spent in bed and more severe fatigue were observed to heighten the risk of a pain flare in low back pain, characterized by a 2-point increase over the average pain [18,19]. These factors collectively contribute to a multitude of impacts, including functional disability, cognitive decline, emotional distress, diminished social interactions, and a lower quality of life [20,21].

Several studies on chronic pain have demonstrated the acceptability, feasibility, and usability of smartphone apps and wearables for remotely monitoring pain severity, disease activity, and associated factors such as sleep patterns, physical activity, mood, and weather conditions [1,3-5]. They have identified specific associations that influence fluctuations in pain severity. For example, certain weather conditions, including higher relative humidity and wind speed and lower atmospheric pressure, were associated with increased pain severity [3]. Worsening symptoms in the preceding week, characterized by higher mean scores and steeper slopes in physical activity, fatigue, sleep difficulty, physical and emotional well-being, and coping ability, were associated with a greater likelihood of self-reported flares [4]. In our exploratory study, we advanced previous research by leveraging daily patient-generated health data captured by mHealth devices to characterize pain flares and identify associated factors in a RA cohort. Our objectives included (1) describing the frequency and duration of pain flares using progressively stringent definitions based on self-reported pain severity, and (2) exploring associations between pain flares and temporal changes in symptoms across emotional, cognitive, and behavioral domains.

## Methods

### mHealth Cohort Study in RA

This secondary analysis used data from a prospective mHealth cohort study that investigated quality of life, sleep, and rheumatoid arthritis (QUASAR) [5]. The QUASAR study recruited 285 participants from the National Rheumatoid Arthritis Society (a UK-wide patient organization) between May 2017 and July 2018. Eligible participants were aged 18 years and older, with a self-reported clinical diagnosis of RA and receiving disease-modifying antirheumatic drugs [22], had access to an Android or iOS smartphone or tablet, and were not employed in shift work. Participants completed a paper-based baseline questionnaire including demographics, information about RA, and their health status. Over the 30-day study period, participants wore a validated triaxial accelerometer (MotionWatch 8 by CamNtech [23-25]) to measure sleep and physical activity. They also used a co-designed smartphone app (uMotif) to complete a daily sleep diary (The 9-item Consensus Sleep Diary, CSD [26]) and provide 10 daily symptoms (pain severity, illness impact, fatigue, mood, well-being, anxiety, disease control, challenge, sleepiness, and concentration) twice a day on a 5-point ordinal scale, once in the morning and once in the evening. Follow-up questionnaires were completed every 10 days after the baseline. A detailed description of the QUASAR study can be found in the protocol [27].

### Data Preparation

#### Participants

A total of 266 participants gave consent for their data to be used for secondary purposes. To be included in the analysis, participants were required to (1) provide symptom data via the study app, (2) provide pain severity data for at least 7 consecutive days, and (3) achieve a completion rate of ≥70% for pain severity data.

#### Baseline Assessments

Demographic data included sex (male or female), age, ethnicity (White or non-White), marital status (single, married or with partner, or separated), occupations (employed, retired, voluntary, or seeking work), smoking status (never, past, or current), and average weekly alcohol consumption (none, moderate: 1‐15 units, and heavy: ≥16 units).

Information about RA included disease duration, menopausal status, other associated rheumatic diseases (osteoarthritis, spondyloarthropathy or ankylosing spondylitis, fibromyalgia or chronic widespread pain, gout or other crystal arthritis, Sjögren syndrome, thyroid disorder, diabetes, multiple sclerosis, and hypertension), sleep-related problems (restless leg syndrome and obstructive sleep apnea or snoring), current medications (sleep medicine and pain medicine), and disease activity, measured by Routine Assessment of Patient Index Data 3 (RAPID-3, score range: 0‐30) [28]. Disease activity was categorized into near remission (≤3), low severity (3.1‐6), moderate severity (6.1‐12), and high severity (≥12).

Health status information consisted of sleep quality, assessed by the Pittsburgh Sleep Quality Index (score range 0‐21, score of >5 indicates poor sleep quality) [29]; insomnia, evaluated by the Sleep Condition Indicator (score range 0‐32, score of ≤16 indicates probable insomnia) [30]; and anxiety and depression, assessed using the Hospital Anxiety and Depression Scale (HADS, score range 0‐21) [31]. HADS was categorized into normal (0‐7), borderline case (8-10), and case (11-21).

#### Pain Severity and Illness Impact

Pain severity and illness impact were scored from 1 (no pain/impact) to 5 (very severe pain/impact) [32]. Higher scores represent greater severity. We used records submitted after 12 PM for data consistency and completeness. For multiple entries within the same day, we selected the final record.

#### Exposure Data

Exposure data consisted of 12 items, including 9 daily self-reported symptoms and 3 objective measurements. All self-reported scores were adjusted to follow the same direction, with higher scores being worse (score range 1‐5). Similar to pain and impact data, only 1 record after 12 PM was included for each day. These daily self-reported symptoms were fatigue (no fatigue to very severe fatigue), mood (very happy to depressed), well-being (very well to very unwell), anxiety (very well to very anxious), disease control (very good control to no control), challenge (not challenging to severely challenging), sleepiness (not sleepy to very sleepy), concentration (excellent to poor), and sleep quality (very good to very poor). Sleep quality was measured by the CSD.

Objective measurements obtained from accelerometer data [33,34] included sleep efficiency (%), sedentary time (%), and time in bed (hour). Sleep efficiency was calculated as the ratio of actual sleep time to total time spent in bed. Sedentary time was defined as the ratio of time spent being sedentary during waking hours. The MotionWatch 8 was configured to sleep mode, capturing limb or bodily movements in 30-second epochs. The raw accelerometer data were processed with MotionWare, an actigraphy software developed by CamNtech. Physical activity during waking hours was calculated by excluding total sleep time and categorizing into 3 segments: sedentary, low, and moderate to vigorous behavior. The cut point of the MotionWatch 8 for sedentary behavior was ≤178.50 counts per minute, while moderate to vigorous activity was set at ≥562.50 counts per minute [35]. Any activity level between these boundaries was recorded as low activity.

### Pain Flare Cycle

A pain flare cycle was divided into 3 phases: preflare, flare, and postflare. The flare was the interval from when pain severity increased until it subsided. Preflare and postflare represented the period of time before and after the flare, respectively.

The onset of a pain flare was defined using a 5-point scale [32,36], including (1) above average (AA): pain severity greater than personal median, (2) above threshold (AT): pain severity greater than 3, and (3) move above threshold (MAT): pain severity moves from 1, 2, or 3 yesterday to either 4 or 5 today. These definitions capture increasingly complex movements in pain levels and demonstrate at least a 25% change, meeting the minimum clinical importance difference [37].

**Figure 1.**
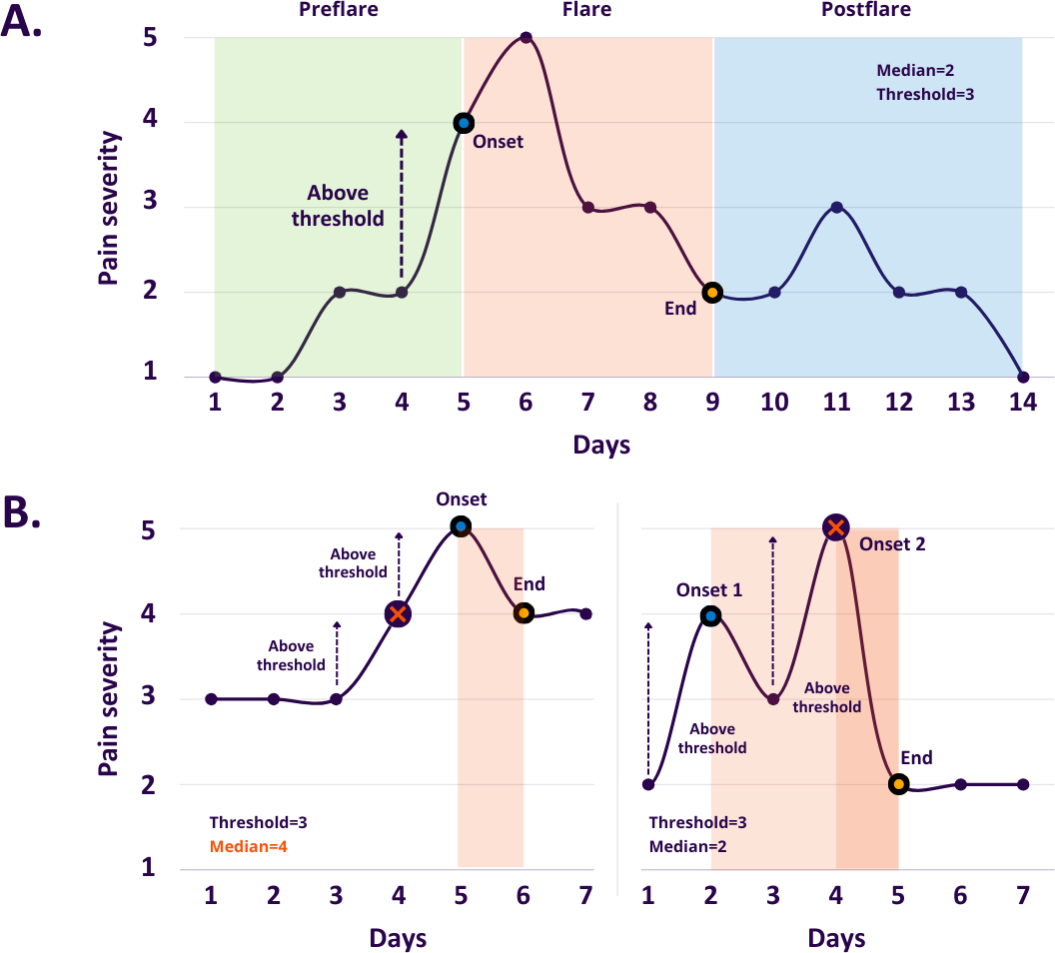
Pain flare identification. The figure shows how pain flares are identified using the definition of above threshold. (A) Pain flare cycle with a median pain score of 2. It includes preflare (green), flare (red), and postflare (blue) phases. The duration of the flare (5 days) is marked between the onset (blue dot) and end (orange dot). (B) Two scenarios of exclusion. Left: the onset (day 4) meets the definition, but the pain severity is at the personal median pain level, conflicting with the criterion for the end of a pain flare. Right: 2 flare onsets (days 2 and 4) meet the definition but end on the same day (day 5). The second onset (day 4) is removed to avoid double counting.

When pain flares occur over multiple consecutive days, the first day of the sequence was identified as the onset of that flare. The end of a pain flare was marked by pain severity returning to the personal median or lower. All flare onsets must occur above the personal median pain scores to avoid conflicts with the criteria defining the end of a flare. When multiple flares end on the same date because of recurring onsets without any return in between, we remove onsets that occurred after the first one to avoid double counting. Figure 1 shows examples of pain flare identification.

#### Pain Flare Impact

In addition to identifying pain flares by their severity, we examined their residual impact in the postflare phase. A carry-on effect was identified for those pain flares where, although pain severity had subsided, the overall illness impact remained elevated above personal median level. The measurement of carry-on effect began from the end of the flare and continued until the illness impact returned to the individual’s median level or below.

#### Hazard and Control Periods

**Figure 2.**
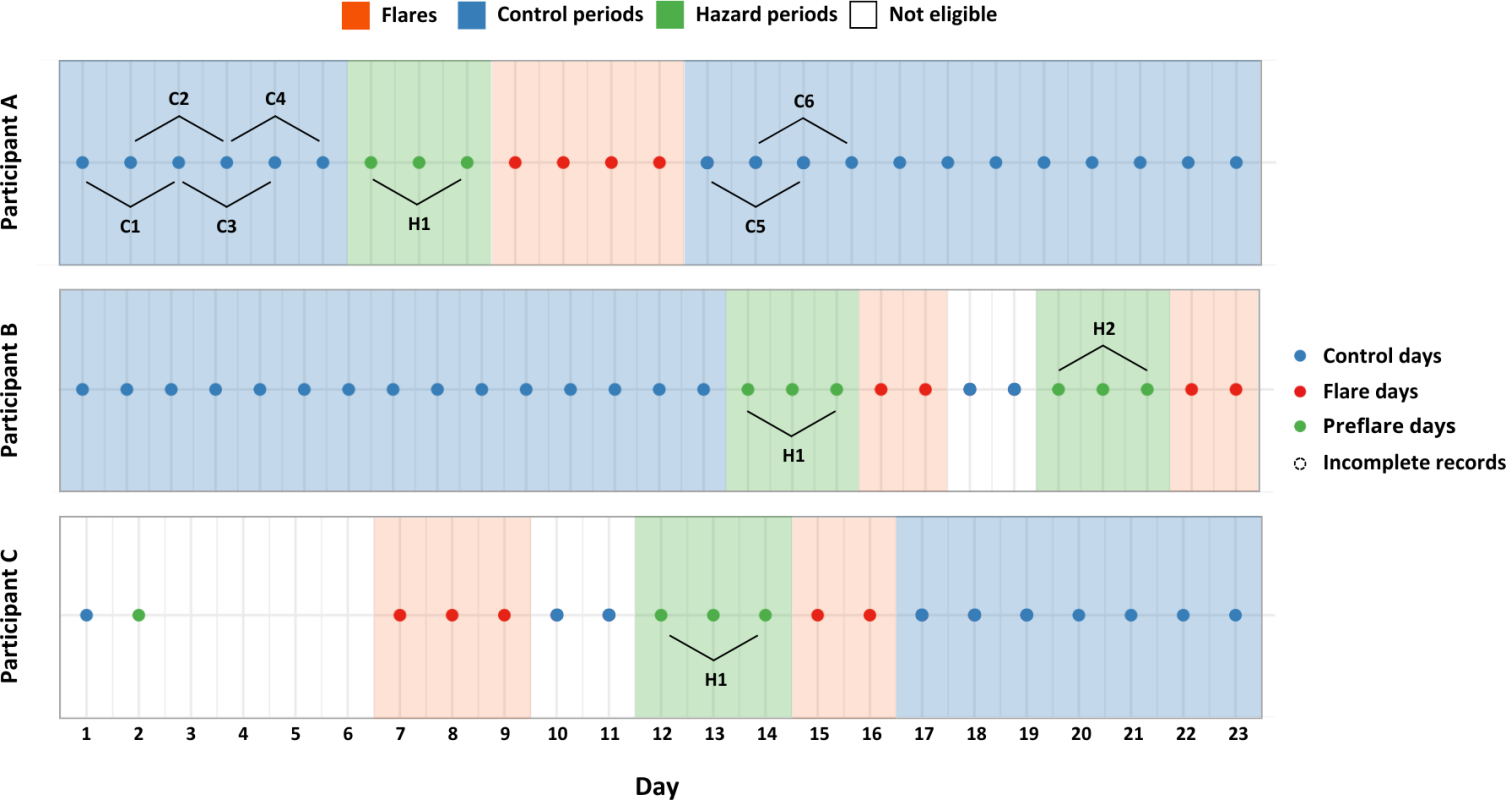
Identification of hazard and control periods. The figure illustrates how hazard and control periods are selected and how risk sets are formed. Hazard periods (green) are 3 days prior to flares (red). Control periods (blue) are 3 days that neither preceded flares (red) nor overlapped with hazard periods (green). A risk set is formed by at least 1 hazard period and 1 control period. Hazard periods (H1 and H2) are compared bidirectionally with control periods (eg, C1, C2, and C3). Ineligible days (white) are excluded from the analysis due to incomplete data.

Hazard periods were selected as the 3 days leading up to each pain flare (preflare phase), excluding any days overlapping with other flares. As a result, not every pain flare was included in the analysis, as it might not have 3 nonoverlapping consecutive preflare days. Control periods were selected using a full stratum bidirectional approach [38] as any 3-day interval that neither preceded a pain flare nor overlapped with other flares or hazard periods, thereby fulfilling the exchangeability assumption for the case-crossover study design [39]. This ensured that there was no overlap between hazard and control periods, and that control periods served as referent windows that were sufficiently recent for comparison and could potentially have been pain flares [40]. Additionally, eligible hazard and control periods must have had complete data for 12 exposures. By using this design, participants serve as their own control, eliminating time-invariant confounders (eg, age, sex, and diagnosis). Each participant with at least 1 hazard and 1 control period formed a risk set. Figure 2 demonstrates the identification of hazard and control periods.

### Statistical Methods

#### Primary Analysis

All analyses were performed using R (version 4.3.1; R Foundation for Statistical Computing). Descriptive analysis was conducted to describe demographics, information about RA, and health status, as well as to characterize pain flares with 3 definitions. For each definition, pain flares were summarized by their frequency, duration, 30-day monthly rate, and the duration of impact.

Conditional logistic regression was used to estimate the odds ratio (OR) for pain flare occurrence. For each pain flare definition, we compared the changes over the 3 days prior to a flare (hazard) with the 3 days that did not precede a flare (control), across a total of 12 exposures. Mean and intraindividual standard deviation (iSD) were used to describe hazard and control periods [41]. The mean represents the average level of each exposure over 3 days. The iSD captures the magnitude of fluctuation over 3 days in each exposure, with higher values indicating increased day-to-day variability. This approach allowed us to determine whether variations in these exposures were associated with pain flares occurring.

Regression models of each definition were analyzed independently. The modeling consisted of 2 steps. First, each of the 12 exposures was analyzed separately using 2 metrics (mean and iSD) in univariable models. This resulted in 24 univariable models, with 2 metrics for each exposure. Subsequently, both metrics for each of the 12 exposures were included in multivariable models, resulting in 12 models. All models were fit using robust sandwich standard errors to account for within-participant correlation due to multiple hazard periods. The findings were presented as ORs with 95% CIs of the likelihood of a pain flare. A 95% CI that does not include the value of 1 indicates a statistically significant association, equivalent to achieving significance as determined by a *P* value.

#### Sensitivity Analysis

Sensitivity analysis was performed to assess the time overlap among control periods. In the primary analysis, control periods were selected as any 3-day interval not preceding a pain flare, during which time overlap was inherently allowed (Figure 2) and could lead to compounding effects of exposures [40,42]. To address this, we conducted a nonoverlap analysis, where control periods were selected as discrete, sequential 3-day intervals (eg, C1 and C4 in Figure 2). This eliminates time overlaps between control periods, thereby avoiding any compounding exposure effect.

### Ethical Considerations

Ethical approval for this secondary analysis was not required. All participants provided consent for secondary use, and all data were fully anonymized. Approval was granted to the QUASAR study [5] by the National Research Ethics Service Committee North West-Liverpool Central Research Ethics Committee (reference: 17/NW/0217).

## Results

### Overview

**Figure 3.**
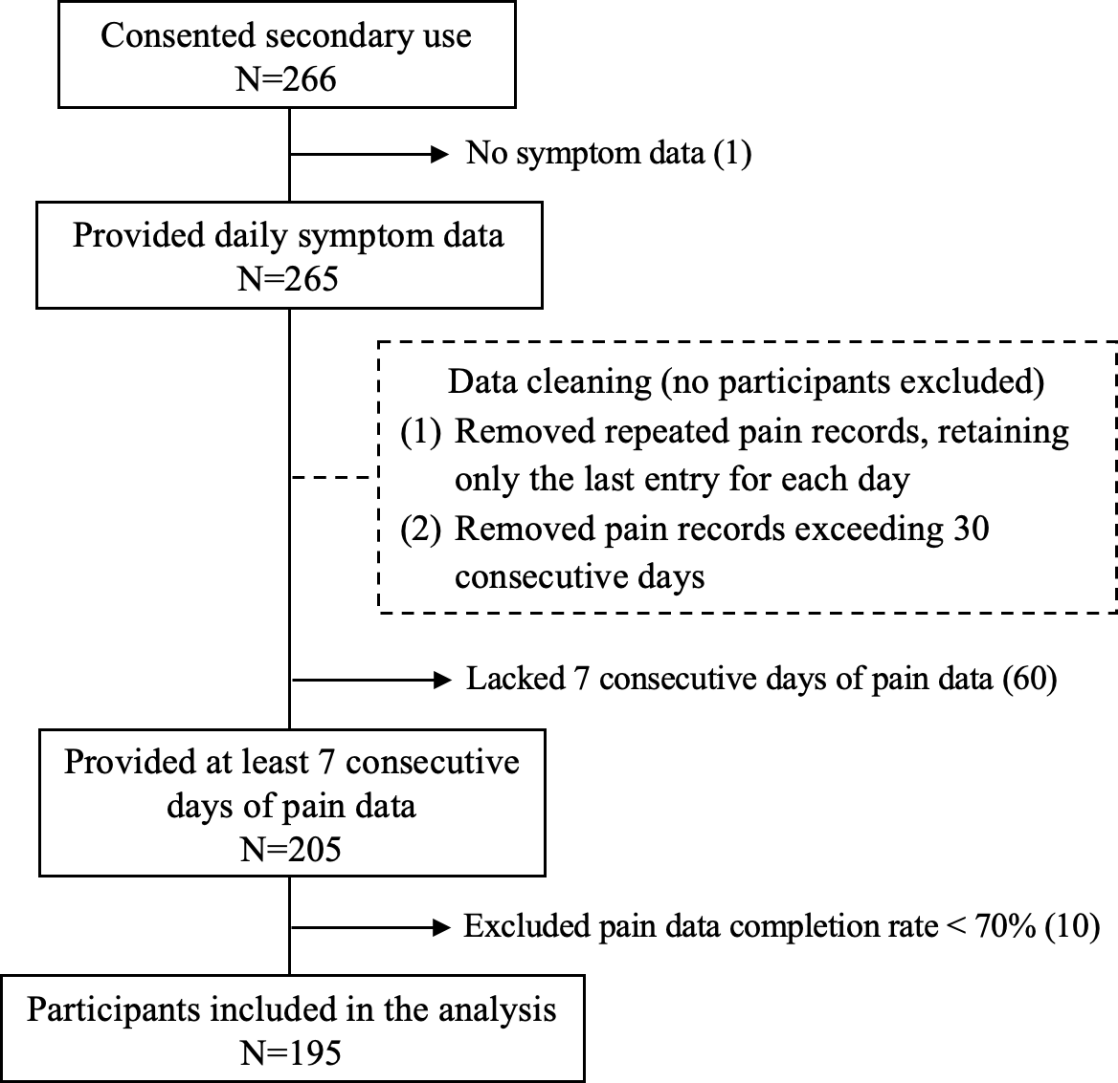
Flow diagram for participant inclusion. The figure shows the participant inclusion process based on three criteria: (1) provides symptom data, (2) provides pain data for at least 7 consecutive days, and (3) achieves a completion rate of ≥70%. The dash-lined box describes the approaches taken to remove duplicates and excessive data, without excluding any participant.

Of the 266 participants who consented for secondary use, 195 (73.3%) were included in the analysis (mean age 57.2 years; mean duration living with RA 11.3 years; 160/195, 82.1% females; 160/195, 82.1% White ethnicity; 92/195, 47.2% employed full time or part-time; and 148/195, 75.9% married or with partner). These participants contributed a total of 5290 person-days of pain data and 3832 person-days of complete exposure data. Figure 3 shows the participant inclusion process. The reasons for exclusion were failing to provide symptom data (n=1) and achieve the required completion rate (n=70).

### Baseline Health Characteristics

Besides RA, 60.5% (118/195) of the participants had one or more coexisting conditions. The most prevalent comorbid condition in this cohort was osteoarthritis (60/195, 30.8%), followed by hypertension (40/195, 20.5%), thyroid disorder (28/195, 14.4%), fibromyalgia (25/195, 12.8%), and Sjögren syndrome (24/195, 12.3%). Most females (119/160, 74.4%) had experienced menopause. Pain medication use was common (157/195, 80.5%). About one-third were also under medical treatment for sleep issues (54/195, 27.7%). More than half had never smoked (107/195, 54.9%) and were consuming moderate alcohol weekly (103/195, 52.8%).

High severity of RA (RAPID-3 median 12.7, IQR 7.5‐17.5) was reported in more than half of the cohort (105/195, 53.8%). Baseline pain level was moderate (median 5.5, IQR 3‐7) on a 11-point scale. Sleep disturbances were common, with 86.7% (169/195) experiencing poor sleep quality (Pittsburgh Sleep Quality Index median 10.3, IQR 7.5‐13.3) and 37.4% (73/195) having probable insomnia (Sleep Condition Indicator median 19, IQR 13‐23). Anxiety cases were reported in 34.4% (67/195) of the cohort (HADS anxiety median 9, IQR 7‐11), whereas 18.5% (36/195) were considered depression cases (HADS depression median 7, IQR 4‐10). Table S1 in Multimedia Appendix 1 provides detailed baseline characteristics.

### Pain Flare Characteristics

As shown in Table 1, the frequency of pain flares decreased when applying stricter definitions. About 88.7% (173/195) of the participants had at least 1 AA flare, with a median monthly rate of 4 flares (IQR 2.1‐5). Nearly half of the participants experienced at least 1 AT (49.2%, 96/195) or MAT (45.6%, 89/195) flare, with a median monthly rate of 2 flares (IQR 1‐4). The average duration of pain flares was consistent across definitions, with a median of 2 days (IQR 2‐3). However, pain flares could last up to 12 days before returning to personal median level or lower.

**Table 1.** Pain flare characteristics.

	Above average	Above threshold	Move above threshold
Participants with ≥1 flare, n (%)	173 (88.7)	96 (49.2)	89 (45.6)
Flare counts, n	662	248	230
Flare duration (days)			
Median (IQR)	2 (2-3)	2 (2-3)	2 (2-3)
Minimum days	2	2	2
Maximum days	12	9	9
30-day monthly rate (flares)			
Median (IQR)	4 (2.1‐5)	2 (1-4)	2 (1-4)
Minimum flares	1	1	1
Maximum flares	9	8	8

### Pain Flare Impact

Analysis of pain flare impact found that fewer than 20% of pain flares across all definitions were affected by carry-on effect (Table 2). In other words, more than 80% of pain flares ended when both their pain severity and illness impact returned to personal median or lower on the same day. Among affected pain flares, a small number were excluded due to either missing impact data or insufficient data time frame. The average duration of these impact-affected pain flares was approximately 4 days but could last up to 12 days. The average duration of impact ranged from as short as 1 day to a week.

**Table 2.** Pain flare impact characteristics.

	Above average	Above threshold	Move above threshold
Total flares affected, n (%)	120 (18.1)	35 (14.1)	34 (14.8)
Total flares analyzed, n	111	34	33
Flare duration (days)			
Median (IQR)	4 (3-5)	4 (3-5)	4 (3-5)
Minimum days	3	3	3
Maximum days	12	11	9
Impact duration (days)			
Median (IQR)	1 (1-2)	1.5 (1‐2.8)	1 (1-2)
Minimum days	1	1	1
Maximum days	7	6	5

### Associations Between Daily Symptoms and Pain Flares

For all definitions, about half of the participants in each subgroup had both hazard and control periods for analysis (Table S2 in Multimedia Appendix 1). As the definitions became stricter, we observed changes in the strength and direction of the relationships between exposures and pain flares. In both univariable and multivariable models, mood demonstrated consistent patterns across all definitions. Mood mean scores were positively associated with pain flare occurrence, whereas iSD scores were negatively associated. Meanwhile, the ORs for sedentary time consistently showed almost no effect, remaining near or at the null value.

### Patient-Reported Exposures

**Figure 4.**
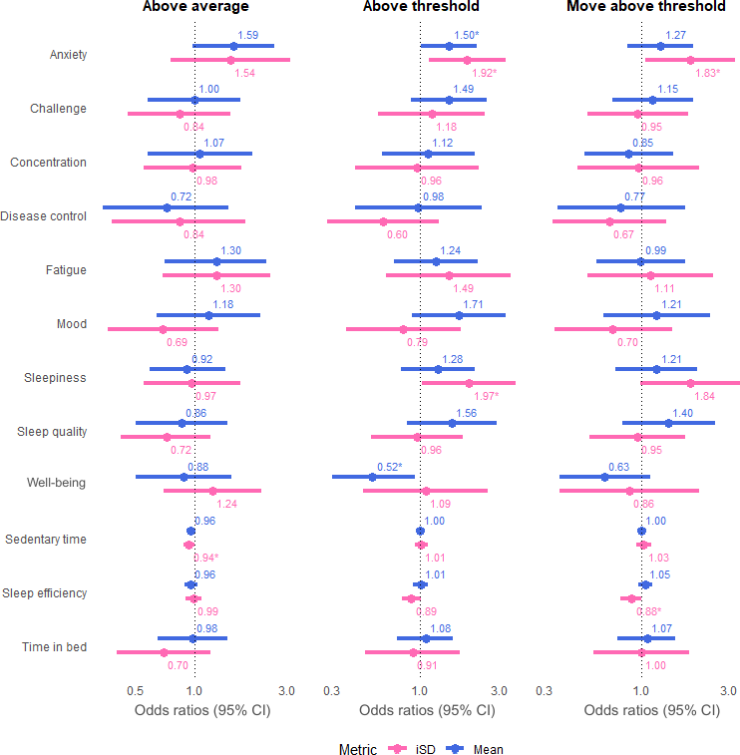
Univariable models across pain flare definitions. The figure shows the results of univariable models across all definitions. Mean (blue) and iSD (pink) were analyzed independently for each of the 12 exposures. *P<.05. iSD: intraindividual standard deviation.

As shown in Figure 4, none of the 9 self-reported symptoms showed a statistically significant relationship with AA flares. However, significant associations were observed for AT and MAT flares. For example, a single unit increase in anxiety mean scores over 3 days was associated with a 1.5 times increased likelihood of an AT flare the following day (OR 1.5, 95% CI 1.02‐2.2). Higher anxiety iSD scores over 3 days were associated with almost a 2-fold increase in the odds of an AT flare the next day (OR 1.92, 95% CI 1.13‐3.27) and a 1.83 times increased likelihood of a MAT flare (OR 1.83, 95% CI 1.05‐3.21). Likewise, higher sleepiness iSD scores over 3 days nearly doubled the odds of an AT flare the next day (OR 1.97, 95% CI 1.03‐3.74). Conversely, higher well-being mean scores reduced the odds of an AT flare by about half (OR 0.52, 95% CI 0.3‐0.93).

**Figure 5.**
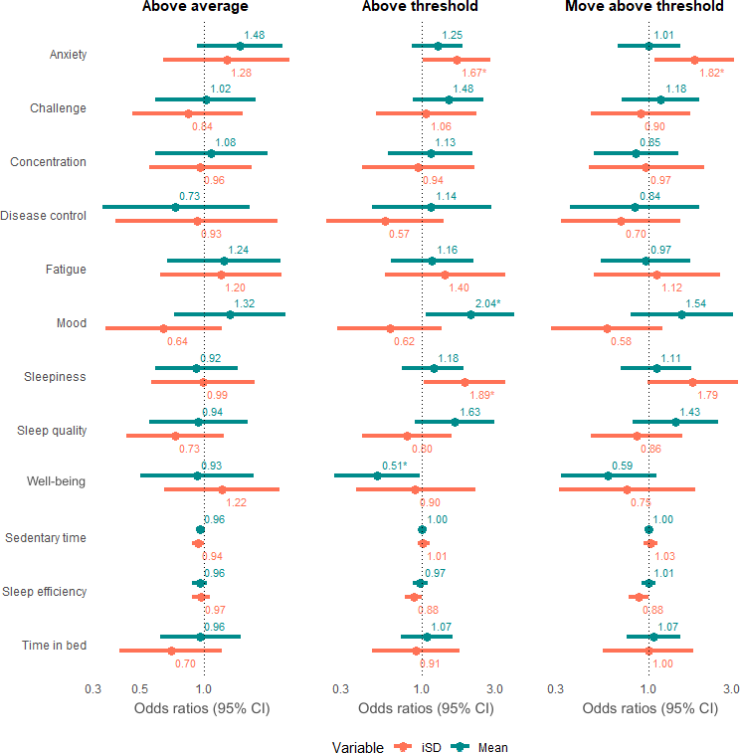
Multivariable models across pain flare definitions. The figure shows the results of multivariable models across all definitions. Both mean (green) and iSD (orange) were included in the analysis for each of the 12 exposures. *P<.05. iSD: intraindividual standard deviation.

In the multivariable models (Figure 5), higher mood mean scores over 3 days were more clearly associated with AT flares, showing a 2-fold increase in the odds after adjusting for the iSD effect (OR 2.04, 95% CI 1.06‐3.94). Higher anxiety iSD scores over 3 days were associated with a 1.67 times increased likelihood of an AT flare the next day (OR 1.67, 95% CI 1.01‐2.78), and a 1.82 times increased likelihood of a MAT flare (OR 1.82, 95% CI 1.08‐3.07). These associations weakened slightly after adjusting for the mean effect. While anxiety mean scores continued to show a positive relationship with AT flares, this association was no longer significant. Well-being mean scores maintained a negative association with AT flares after adjusting for the iSD effect, reducing the odds by nearly half (OR 0.51, 95% CI 0.27‐0.97). See full results in Tables S3 and S4 in Multimedia Appendix 1.

. .

### Accelerometer-Derived Exposures

All objective measurements, including sedentary time, sleep efficiency, and time in bed, demonstrated no significant associations with pain flares across definitions in both univariable and multivariable models (Figures4  5). The majority of associations were near or at an OR of 1, even after adjustments were made for the mean and iSD effects. Full results are shown in Tables S3 and S4 in Multimedia Appendix 1.

### Sensitivity Analysis

**Figure 6.**
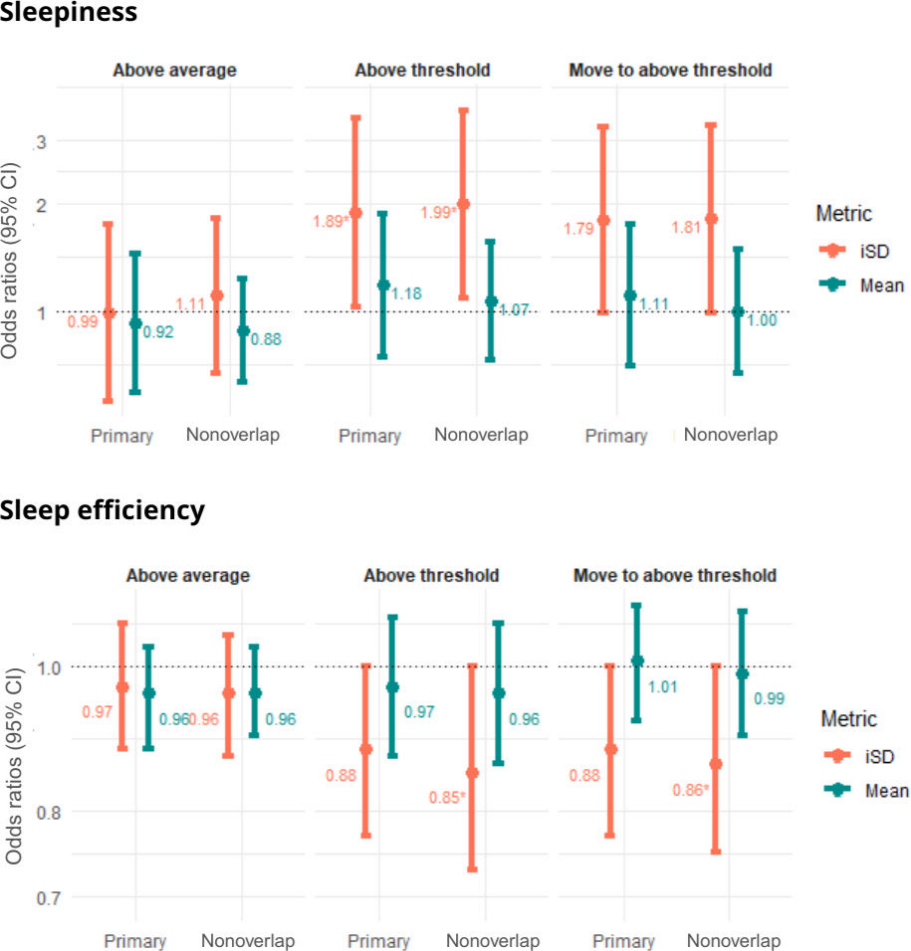
Sensitivity analysis results for primary and nonoverlap models. The figure shows sensitivity analysis results for primary and nonoverlap models of sleepiness and sleep efficiency. The multivariable model includes mean (green) and iSD (orange). *P<.05. iSD: intraindividual standard deviation.

The results of the nonoverlap analysis were similar to those of the primary analysis, with most displaying narrower confidence intervals. However, variations in direction and strength of associations were observed. Across primary and nonoverlap analyses, no significant association was observed in AA flares. As shown in Figure 6, higher sleepiness iSD scores over 3 days were consistently associated with a 2-fold increase in the odds of an AT flare the next day, with an OR of 2.04 (95% CI 1.13‐3.71) in the univariable model and 1.99 (95% CI 1.09‐3.63) after adjusting for the mean effect.

We observed a negative relationship between well-being mean scores and AT flares (OR 0.58, 95% CI 0.35‐0.98), but this association did not persist after adjusting for the iSD effect. Similarly, higher sleep efficiency iSD scores reduced the likelihood of a MAT flare the following day (OR 0.87, 95% CI 0.76‐0.99) but did not persist after adjusting the mean effect. Full results are shown in Figures S1 and S2 in Multimedia Appendix 1.

## Discussion

### Principal Findings

This exploratory study demonstrates the potential of mHealth technologies to improve our understanding of pain fluctuations in RA. By leveraging daily patient-generated health data, it was possible to describe the frequency and duration of pain flares and identify within-person associated factors. These findings would be difficult to obtain through traditional assessments. However, achieving this level of understanding also relied on sustained patient engagement with the technology, which is challenging due to various barriers such as user fatigue, technology literacy, or motivation [43]. The loss of engagement can substantially impact the quality of data, leading to biases, misinterpretation, and incorrect conclusions. The QUASAR study, achieving near 90% engagement [5], provides a strong foundation for our analysis. The results led to 3 novel observations regarding pain flares and implications for future pain research using mHealth technologies. Additionally, we discuss limitations that need to be addressed for future improvement.

First, using 3 numerically defined pain flares, we found that the average duration consistently spans 2 days across all definitions. When considering their overall impact, the average duration extends to 4 days for all definitions. This suggests that, regardless of the sudden increase in pain severity, the initial surge typically diminishes within a few days; however, the reduction in pain severity does not necessarily indicate the end of its impact. Qualitative studies have documented the experience of flare-ups as highly individualized and complex [11,20]. Pain flares cannot be fully explained by pain levels alone; their broader impact must also be considered.

Second, emotional distress, as measured by mood and anxiety, is closely associated with pain flares. Feeling more depressed over 3 days appears to increase the likelihood of experiencing a pain flare the following day, consistent with previous research indicating that higher levels of depression are associated with increased pain [16]. Conversely, experiencing a higher degree of mood changes seems to reduce the likelihood of pain flare occurrence. This suggests that varied mood fluctuations could represent normal mood regulation, rather than being in a heightened depressive state. Contrary to previous findings indicating no predictive relationship between anxiety and pain variability [16], our findings show that feeling more anxious and experiencing more fluctuations in anxiety over 3 days increase the likelihood of pain flares.

Finally, patient-reported and accelerometer-derived measurements of sleep demonstrate varied relationships with pain flares. Perceived sleep quality shows no noticeable associations, regardless of the definition used. Perceived sleepiness shows a positive association with pain flares, except under the simplest definition where it has nearly no influence. A positive association was observed between longer time spent in bed and an increased likelihood of pain flares, a pattern similarly noted in low back pain [19]. However, none of these associations were statistically significant. Sleep efficiency demonstrates almost no influence on pain flares across definitions. It is worth noting that self-reported sleep patterns did not strongly correlate with actigraphy-derived sleep patterns in the QUASAR study [5]. Perceived and objective sleep measurements may capture different aspects of sleep issues. For example, perceived sleepiness likely reflects the cumulative impact of long-term poor sleep, whereas sleep efficiency could indicate short-term fluctuations in sleep patterns. The choice between subjective or objective sleep measures needs to be guided by their clinical relevance to pain management and tailored to the individual needs, a process that mHealth technologies can facilitate by integrating data from multiple sources to provide a more holistic view of sleep patterns.

The remaining symptoms, including perceived fatigue, concentration, disease control, and challenge, did not exhibit consistent patterns across definitions. Their temporal changes may not be directly associated with the occurrence of pain flares but might instead be more salient in their impact afterward. Sedentary time demonstrates minimal to no associations with pain flares. A similar pattern was also observed in prior research on low back pain, which found that longer sedentary behavior did not increase the odds of pain flares the next day [19]. An unexpected association was observed for well-being, where feeling more unwell appeared to lower the likelihood of pain flare occurrence. This counterintuitive finding needs further investigation, as changes in emotional state could alter pain management behaviors and potentially influence the pain experience [44].

### Study Strengths and Limitations

The study strengths include using a large sample size with more than 15% of participants from diverse ethnic backgrounds, examining across multiple numerically defined pain flares, and employing case-crossover design, which collectively enhance the robustness of capturing within-person variations over time. Several limitations need to be highlighted.

First, the nature of RA as an inflammatory disease may complicate the definition of pain flares. Our approach using pain severity alone may overlook other factors contributing to increased pain, such as inflammation or medication changes. Second, despite using 3 definitions to encompass diverse pain fluctuations, participants with consistently mild or severe pain may not have been included in the analysis because their lack of fluctuations failed to meet any of the definitions. Third, we selected a 3-day window for hazard and control periods and adopted a full stratum bidirectional approach to maximize data availability. This approach was chosen to account for the constraints of the 30-day study period while minimizing overlaps between hazard periods and pain flares. However, there were overlaps between control periods and postflare phases, during which symptoms could persist and potentially intertwine with the carry-on effects of pain flares. Excluding postflare phases and using alternative options, such as a 7-day window, were considered but deemed unsuitable, given the data availability. Our sensitivity analysis supports the current approach as a robust method. Employing a rolling window to capture fluctuations in exposures reflects real-world dynamics. After adjusting for these overlaps, we observed the same patterns in exposure outcome associations with expected variations due to changes in statistical power. Fourth, physical activity was not quantified based on individual calibration, and the accelerometer was optimized to record sleep data, potentially compromising the sensitivity in detecting movements during daytime. Finally, days with missing data for pain or any of the 12 exposures were excluded. This likely introduced selection bias, reducing the number of participants included in the analysis and limiting the availability of hazard and control periods. Imputation was not used to avoid introducing bias, as the missing data were likely not missing at random.

### Implications and Future Research

The study provides a novel narrative in understanding pain flares through daily patient-generated data. It shows the potential of mHealth technologies for multidimensional monitoring of symptom patterns. This approach enables a more nuanced understanding of individual pain experiences and associated factors, which offers health care providers and patients an opportunity to better predict and proactively manage pain flares [45]. Incorporating both patient-reported and accelerometer-derived measures allows for capturing a more complete view of a patient’s condition and its progression. It also nudges active patient involvement in their own care, promoting self-monitoring to increase awareness. However, it is important to recognize that patient engagement with mHealth technologies remains a significant challenge in digital health research, with issues such as data missingness affecting the quality of the data or lack of transparency compromising the integrity of reporting. Digital exclusion, contributed by factors such as socioeconomic disparities or lack of digital skills, is another critical factor that could limit the generalizability of digital health research. The QUASAR study, for example, excluded individuals who did not own a personal smartphone or tablet. This restriction may have inadvertently overlooked certain populations and their unique characteristics.

For pain research specifically, future studies are recommended to include multiple pain-related symptoms and explore how symptoms vary during different stages of pain flares. This approach will enhance our understanding of their nuances and impact on patients, potentially leading to more timely monitoring and personalized management strategies. Furthermore, future studies also need to consider the impact of digital exclusion and strive for greater inclusivity to encourage wider participation.

### Conclusions

Our study, which leveraged mHealth technologies and numerically defined pain severity, has concluded that pain flares are commonly observed in patients with RA. The analysis of daily patient-generated health data indicates that changes in sleep patterns and emotional distress over 3 days may be associated with the occurrence of pain flares the following day. This study represents an early example of identifying pain flares using daily data, opening opportunities for timely monitoring and personalized management. As digital technologies evolve, they hold significant potential to transform how we understand and manage chronic conditions. It is also crucial to address the challenges of patient engagement, the impact of digital exclusion, and the need for greater inclusivity in future research.

## Supplementary material

10.2196/64889 Multimedia Appendix 1 Supplementary materials supporting the results.

## Abbreviations

AA: above average
AT: above threshold
CSD: Consensus Sleep Diary
HADS: Hospital Anxiety and Depression Scale
iSD: intraindividual standard deviation
MAT: move above threshold
mHealth: mobile health
OR: odds ratio
QUASAR: quality of life, sleep, and rheumatoid arthritis
RA: rheumatoid arthritis
RAPID-3: Routine Assessment of Patient Index Data 3
