# Supplementary material for: Associations Between Daily Symptoms and Pain Flares in Rheumatoid Arthritis: Case-Crossover mHealth Study
Source: JMIR Mhealth Uhealth. 2025 Jul 21;13:e64889. doi: 10.2196/64889 (PMC12303358; doi:10.2196/64889)

Table S1. Characteristics of the study cohort.

| **Characteristics** | **Included participants (N = 195)** |
| --- | --- |
| **Female, n (%)** | 160 (82.1) |
| **Age** | |
| Median (IQR) | 58.2 (51-65.2) |
| Minimum age | 26.7 |
| Maximum age | 79.6 |
| **Ethnicity, n (%)** | |
| White* | 160 (82.1) |
| Mixed | 1 (0.5) |
| Asian | 1 (0.5) |
| Black, African, Caribbean | 1 (0.5) |
| Others** | 32 (16.4) |
| **Occupations, n (%)** | |
| Employed (full-time, part-time) | 92 (47.2) |
| Retired (medically retired, retired) | 90 (46.2) |
| Others (voluntary worker, seeking work) | 12 (6.2) |
| Missing | 1 (0.5) |
| **Marital status, n (%)** | |
| Single | 11 (5.6) |
| Married or with partner (civil partnership, in a relationship, co-habiting) | 148 (75.9) |
| Divorced, separated, widowed | 35 (18) |
| Missing | 1 (0.5) |
| **Rheumatoid arthritis, median (IQR)** | |
| Disease duration (years) | 10.1 (4.8-16.3) |
| Disease activity (RAPID3, range 0-30) | 12.7 (7.5-17.5) |
| Baseline pain (RAPID3, range 0-10) | 5.5 (3-7) |
| **Number of comorbidities, n (%)** | |
| None | 77 (39.5) |
| 1 | 48 (24.6) |
| 2 | 38 (19.5) |
| 3 or more | 32 (16.4) |
| **Have menopause (female), n (%)** | 119 (74.4) |
| **Medications, n (%)** | |
| Take sleeping medicine | 54 (27.7) |
| Take pain medicine | 157 (80.5) |
| **Sleep status** | |
| Sleep quality (PSQI, range 0-21), median (IQR) | 10.3 (7.5-13.3) |
| Poor sleep quality (PSQI > 5), n (%) | 169 (86.7) |
| Insomnia (SCI, range 0-32), median (IQR) | 19 (13-23) |
| Possible insomnia (SCI ≤ 16), n (%) | 73 (37.4) |
| Have restless leg syndrome, n (%) | 18 (9.2) |
| Have obstructive sleep apnea, n (%) | 12 (6.2) |
| **Anxiety (HADS subscale), n (%)** | |
| Normal (1-7) | 65 (33.3) |
| Borderline case (8-10) | 63 (32.3) |
| Case (11-21) | 67 (34.4) |
| **Depression (HADS subscale), n (%)** |  |
| Normal (0-7) | 109 (55.9) |
| Borderline case (8-10) | 50 (25.6) |
| Case (11-21) | 36 (18.5) |
| **Smoking status, n (%)** |  |
| Ever smoke (current, ex) | 85 (43.6) |
| Never smoke | 107 (54.9) |
| Missing | 3 (1.5) |
| **Alcohol consumption, n (%)** |  |
| None | 77 (39.5) |
| Moderate (1-15 unit) | 103 (52.8) |
| Heavy (≥ 16 unit) | 14 (7.2) |
| Missing | 1 (0.5) |

Note: * Caucasian, British, English, Scottish, Irish, European; **Including unspecified British, English, Welsh, Scottish, Irish, Northern Irish

Table S2. Summary of pain flares, hazard, and control periods.

|  | **Above average** | **Above threshold** | **Move above threshold** |
| --- | --- | --- | --- |
| **Total participants (N = 195)** | | | |
| Participants with ≥ 1 pain flare, N (%) | 173 (88.7) | 96 (49.2) | 89 (45.6) |
| Pain flare counts, N | 662 | 248 | 230 |
| Participants had risk sets*, N (%) | 88 (50.9) | 47 (49.0) | 51 (57.3) |
| Pain flare counts, N | 325 | 122 | 138 |
| Hazard counts, N | 130 | 63 | 71 |
| Control counts, N | 495 | 368 | 389 |
| Non-overlap control counts, N | 223 | 156 | 167 |

Note: *a risk set comprises at least one hazard period and one control period

Table S3. Primary univariable models.

|  | | **Above average** | | **Above threshold** | | **Move above threshold** | |
| --- | --- | --- | --- | --- | --- | --- | --- |
|  |  | OR (95% CI) | p value | OR (95% CI) | p value | OR (95% CI) | p value |
| **Subjective measures** | | | | | | | |
| Anxiety | Mean | 1.59 (0.98-2.58) | 0.06 | **1.50 (1.02-2.20)** | **0.04** | 1.27 (0.84-1.90) | 0.25 |
|  | iSD | 1.54 (0.76-3.12) | 0.23 | **1.92 (1.13-3.27)** | **0.02** | **1.83 (1.05-3.21)** | **0.03** |
| Challenge | Mean | 1.00 (0.58-1.72) | 0.99 | 1.49 (0.89-2.52) | 0.13 | 1.15 (0.70-1.90) | 0.58 |
|  | iSD | 0.84 (0.46-1.53) | 0.57 | 1.18 (0.56-2.46) | 0.66 | 0.95 (0.51-1.79) | 0.89 |
| Concentration | Mean | 1.07 (0.58-2.00) | 0.82 | 1.12 (0.59-2.13) | 0.72 | 0.85 (0.49-1.49) | 0.58 |
|  | iSD | 0.98 (0.55-1.75) | 0.94 | 0.96 (0.41-2.24) | 0.92 | 0.96 (0.45-2.06) | 0.91 |
| Disease control | Mean | 0.72 (0.34-1.50) | 0.38 | 0.98 (0.41-2.34) | 0.97 | 0.77 (0.35-1.72) | 0.53 |
|  | iSD | 0.84 (0.38-1.84) | 0.66 | 0.60 (0.28-1.30) | 0.19 | 0.67 (0.33-1.37) | 0.27 |
| Fatigue | Mean | 1.30 (0.71-2.35) | 0.39 | 1.24 (0.70-2.21) | 0.46 | 0.99 (0.57-1.73) | 0.98 |
|  | iSD | 1.30 (0.69-2.47) | 0.42 | 1.49 (0.63-3.51) | 0.36 | 1.11 (0.51-2.43) | 0.80 |
| Mood | Mean | 1.18 (0.64-2.18) | 0.60 | 1.71 (0.90-3.26) | 0.10 | 1.21 (0.62-2.35) | 0.57 |
|  | iSD | 0.69 (0.36-1.33) | 0.27 | 0.79 (0.36-1.76) | 0.57 | 0.70 (0.34-1.46) | 0.34 |
| Sleepiness | Mean | 0.92 (0.59-1.45) | 0.73 | 1.28 (0.77-2.13) | 0.35 | 1.21 (0.72-2.01) | 0.47 |
|  | iSD | 0.97 (0.55-1.72) | 0.92 | **1.97 (1.03-3.74)** | **0.04** | 1.84 (0.99-3.44) | 0.06 |
| Sleep quality | Mean | 0.86 (0.50-1.49) | 0.59 | 1.56 (0.84-2.90) | 0.16 | 1.40 (0.79-2.51) | 0.25 |
|  | iSD | 0.72 (0.42-1.21) | 0.21 | 0.96 (0.51-1.80) | 0.90 | 0.95 (0.52-1.72) | 0.86 |
| Wellbeing | Mean | 0.88 (0.50-1.56) | 0.67 | **0.52 (0.30-0.93)** | **0.03** | 0.63 (0.36-1.12) | 0.11 |
|  | iSD | 1.24 (0.70-2.22) | 0.46 | 1.09 (0.46-2.56) | 0.84 | 0.86 (0.36-2.05) | 0.73 |
| **Objective measures** | | | | | | | |
| Sedentary time | Mean | 0.96 (0.92-1.00) | 0.05 | 1.00 (0.95-1.07) | 0.90 | 1.00 (0.95-1.06) | 0.99 |
|  | iSD | 0.94 (0.88-1.00) | **0.04** | 1.01 (0.93-1.11) | 0.77 | 1.03 (0.94-1.13) | 0.49 |
| Sleep efficiency | Mean | 0.96 (0.89-1.04) | 0.34 | 1.01 (0.91-1.12) | 0.88 | 1.05 (0.96-1.14) | 0.30 |
|  | iSD | 0.99 (0.90-1.09) | 0.84 | 0.89 (0.78-1.02) | 0.10 | 0.88 (0.77-1.00) | **0.04** |
| Time in bed | Mean | 0.98 (0.65-1.48) | 0.92 | 1.08 (0.73-1.58) | 0.71 | 1.07 (0.74-1.53) | 0.72 |
|  | iSD | 0.70 (0.40-1.21) | 0.20 | 0.91 (0.47-1.74) | 0.77 | 1.00 (0.55-1.81) | 0.99 |

Note: bold – p < 0.05

Table S4. Primary multivariable models.

|  | | **Above average** | | **Above threshold** | | **Move above threshold** | |
| --- | --- | --- | --- | --- | --- | --- | --- |
|  |  | OR (95% CI) | p value | OR (95% CI) | p value | OR (95% CI) | p value |
| **Subjective measures** | | | | | | | |
| Anxiety | Mean | 1.48 (0.93-2.36) | 0.10 | 1.25 (0.86-1.82) | 0.25 | 1.01 (0.67-1.53) | 0.95 |
|  | iSD | 1.28 (0.64-2.55) | 0.49 | **1.67 (1.01-2.78)** | **0.05** | **1.82 (1.08-3.07)** | **0.03** |
| Challenge | Mean | 1.02 (0.59-1.76) | 0.94 | 1.48 (0.87-2.50) | 0.14 | 1.18 (0.71-1.96) | 0.51 |
|  | iSD | 0.84 (0.46-1.53) | 0.57 | 1.06 (0.50-2.23) | 0.88 | 0.90 (0.47-1.74) | 0.76 |
| Concentration | Mean | 1.08 (0.59-2.00) | 0.80 | 1.13 (0.60-2.12) | 0.70 | 0.85 (0.49-1.49) | 0.58 |
|  | iSD | 0.96 (0.55-1.68) | 0.88 | 0.94 (0.41-2.18) | 0.89 | 0.97 (0.46-2.07) | 0.94 |
| Disease control | Mean | 0.73 (0.33-1.64) | 0.45 | 1.14 (0.47-2.80) | 0.77 | 0.84 (0.36-1.96) | 0.69 |
|  | iSD | 0.93 (0.38-2.24) | 0.86 | 0.57 (0.24-1.37) | 0.21 | 0.70 (0.32-1.53) | 0.37 |
| Fatigue | Mean | 1.24 (0.67-2.30) | 0.49 | 1.16 (0.63-2.14) | 0.64 | 0.97 (0.54-1.73) | 0.92 |
|  | iSD | 1.20 (0.62-2.32) | 0.59 | 1.40 (0.57-3.48) | 0.46 | 1.12 (0.49-2.55) | 0.78 |
| Mood | Mean | 1.32 (0.72-2.43) | 0.37 | **2.04 (1.06-3.94)** | **0.03** | 1.54 (0.79-3.02) | 0.21 |
|  | iSD | 0.64 (0.34-1.22) | 0.18 | 0.62 (0.28-1.34) | 0.22 | 0.58 (0.28-1.21) | 0.15 |
| Sleepiness | Mean | 0.92 (0.59-1.44) | 0.73 | 1.18 (0.74-1.86) | 0.49 | 1.11 (0.70-1.75) | 0.65 |
|  | iSD | 0.99 (0.56-1.74) | 0.98 | **1.89 (1.03-3.47)** | **0.04** | 1.79 (0.99-3.26) | 0.06 |
| Sleep quality | Mean | 0.94 (0.55-1.62) | 0.83 | 1.63 (0.90-2.94) | 0.11 | 1.43 (0.82-2.51) | 0.21 |
|  | iSD | 0.73 (0.43-1.24) | 0.24 | 0.80 (0.41-1.54) | 0.50 | 0.86 (0.47-1.56) | 0.62 |
| Wellbeing | Mean | 0.93 (0.50-1.72) | 0.82 | **0.51 (0.27-0.97)** | **0.04** | 0.59 (0.32-1.11) | 0.10 |
|  | iSD | 1.22 (0.65-2.28) | 0.54 | 0.90 (0.37-2.22) | 0.82 | 0.75 (0.31-1.84) | 0.53 |
| **Objective measures** | | | | | | | |
| Sedentary time | Mean | 0.96 (0.92-1.00) | 0.06 | 1.00 (0.95-1.07) | 0.89 | 1.00 (0.95-1.06) | 0.99 |
|  | iSD | 0.94 (0.88-1.00) | 0.07 | 1.01 (0.93-1.11) | 0.77 | 1.03 (0.94-1.13) | 0.49 |
| Sleep efficiency | Mean | 0.96 (0.88-1.03) | 0.26 | 0.97 (0.87-1.08) | 0.53 | 1.01 (0.92-1.10) | 0.90 |
|  | iSD | 0.97 (0.88-1.07) | 0.51 | 0.88 (0.77-1.00) | 0.05 | 0.88 (0.77-1.00) | 0.06 |
| Time in bed | Mean | 0.96 (0.62-1.49) | 0.87 | 1.07 (0.73-1.58) | 0.72 | 1.07 (0.75-1.53) | 0.72 |
|  | iSD | 0.70 (0.40-1.21) | 0.20 | 0.91 (0.47-1.75) | 0.78 | 1.00 (0.55-1.80) | 0.99 |

Note: bold - p < 0.05

Figure S1. Forest plots of non-overlap univariable models.


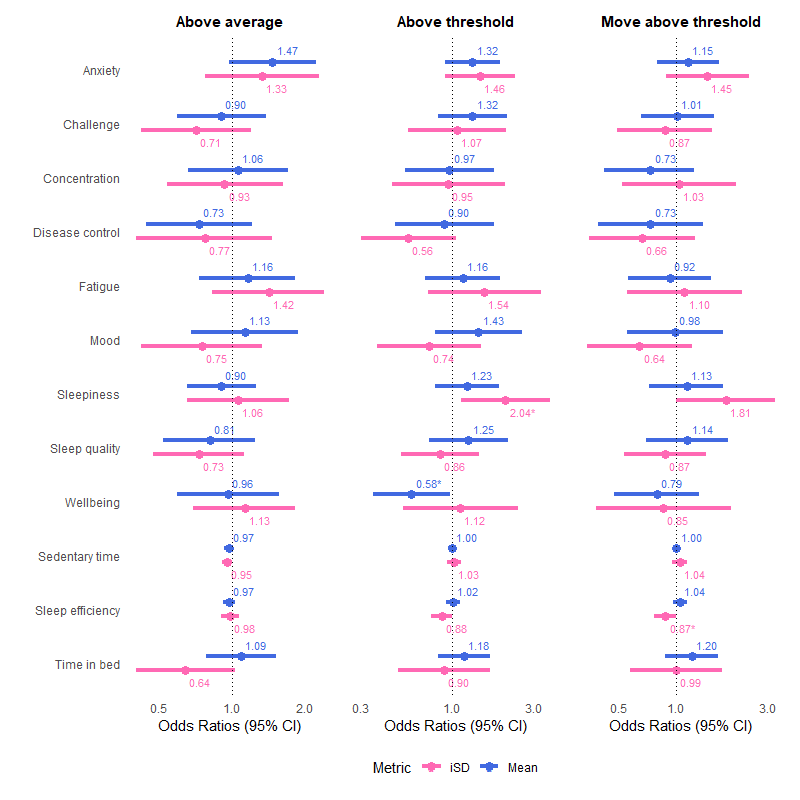


Figure S2. Forest plots of non-overlap multivariable models.


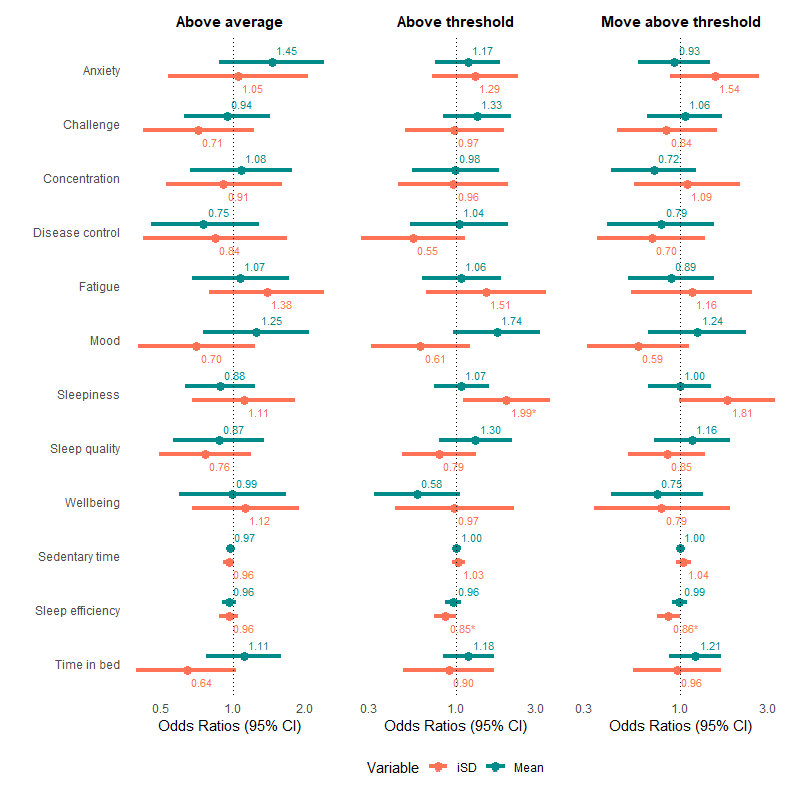

Supplement: Multimedia Appendix 1 [file mhealth-v13-e64889-s001.docx]
